# Neuromagnetic Indicators of Tinnitus and Tinnitus Masking in Patients with and without Hearing Loss

**DOI:** 10.1007/s10162-012-0340-5

**Published:** 2012-07-12

**Authors:** Peyman Adjamian, Magdalena Sereda, Oliver Zobay, Deborah A. Hall, Alan R. Palmer

**Affiliations:** 1 MRC Institute of Hearing Research, University Park, Nottingham, UK NG7 2RD; 2 NIHR Nottingham Hearing Biomedical Research Unit, Nottingham, UK NG1 5DU

**Keywords:** thalamocortical dysrhythmia, tinnitus masking, magnetoencephalography, cortical oscillations

## Abstract

Tinnitus is an auditory phenomenon characterised by the perception of a sound in the absence of an external auditory stimulus. Chronic subjective tinnitus is almost certainly maintained via central mechanisms, and this is consistent with observed measures of altered spontaneous brain activity. A number of putative central auditory mechanisms for tinnitus have been proposed. The influential thalamocortical dysrhythmia model suggests that tinnitus can be attributed to the disruption of coherent oscillatory activity between thalamus and cortex following hearing loss. However, the extent to which this disruption specifically contributes to tinnitus or is simply a consequence of the hearing loss is unclear because the necessary matched controls have not been tested. Here, we rigorously test several predictions made by this model in four groups of participants (tinnitus with hearing loss, tinnitus with clinically normal hearing, no tinnitus with hearing loss and no tinnitus with clinically normal hearing). Magnetoencephalography was used to measure oscillatory brain activity within different frequency bands in a ‘resting’ state and during presentation of a masking noise. Results revealed that low-frequency activity in the delta band (1–4 Hz) was significantly higher in the ‘tinnitus with hearing loss’ group compared to the ‘no tinnitus with normal hearing’ group. A planned comparison indicated that this effect was unlikely to be driven by the hearing loss alone, but could possibly be a consequence of tinnitus and hearing loss. A further interpretative linkage to tinnitus was given by the result that the delta activity tended to reduce when tinnitus was masked. High-frequency activity in the gamma band (25–80 Hz) was not correlated with tinnitus (or hearing loss). The findings partly support the thalamocortical dysrhythmia model and suggest that slow-wave (delta band) activity may be a more reliable correlate of tinnitus than high-frequency activity.

## Introduction

Tinnitus (TI) is the perception of sound in the absence of an external source. Approximately 10 % of the population experience TI permanently, and in around 1–3 % of these people, their quality of life is severely affected by the condition (Davis and El Rafaie 2000). The current paper is concerned with chronic subjective TI, which can be heard only by the sufferer (i.e. there is no physical explanation for the perceived sound). A general assumption from animal models of TI is that the condition is due to abnormal activity in the central auditory system, as a consequence of altered patterns of neural activity following a measurable hearing impairment (Eggermont and Roberts 2004). However, we acknowledge that this is just one of several explanations because not all people with hearing loss experience TI and many people with TI have clinically normal hearing (Schaette and McAlpine 2011; Weisz et al. 2006).

One of the more influential models within the general field of neuroscience has been the thalamocortical dysrhythmia model, which considers the reciprocal circuits between thalamus and cortex to be fundamental to a range of positive/negative symptomatology including not only TI but also neurogenic pain, depression, and Parkinson’s disease (Llinas et al. 1999). The model posits that TI is the consequence of disruption of coherent activity between thalamus and cortex following hearing loss. It postulates that abnormalities arise from neural events that are initiated by input deafferentation and cause ‘overinhibition’ of thalamic neurons reducing their excitatory drive. The loss of excitatory inputs results in the production of bursts of low-threshold calcium spikes and the hyperpolarisation of membrane potentials of thalamic neurons. This thalamic overinhibition leads to large-scale slow-wave coherence that activates the return thalamocortical pathways and entrains the circuit into delta/theta oscillatory activity. At the cortical level, the focal slow-wave oscillations of cortico-cortical inhibitory interneurons reduce lateral inhibition and disinhibit beta and gamma oscillations in neighbouring cortical regions, giving rise to an ‘edge effect’ (Llinas et al. 2005). In summary, therefore, the intact thalamocortical circuit is proposed to oscillate in the gamma frequency band (∼40 Hz), while the deafferented circuit is proposed to oscillate in the theta band (4–8 Hz). Thus, while slow-wave activity is the signature of neural deafferentation in cases of TI associated with hearing loss, the model appears to suggest that gamma activity is the correlate of the TI percept itself (Llinas et al. 1999, 2005). High-frequency hearing loss may not be a necessary prerequisite for increased gamma band activity because, in the normal brain, gamma-band oscillatory activity increases when an external sound is present (Joliot et al. 1994; Crone et al. 2001). This finding raises the possibility that TI without hearing loss may be associated with abnormal gamma activity.

The theory of Llinas et al. makes a number of specific predictions that can be tested in humans using electroencephalography (EEG) or magnetoencephalography (MEG): (1) in the presence of hearing loss, TI should be associated with an increase in oscillatory activity in low frequency (<10 Hz) and high-frequency (gamma) bands (25–50 Hz), and (2) altered gamma activity should be maximal at the edge of the hearing loss in primary auditory cortex (the ‘edge effect’). The first prediction can be tested either in a within-subject design (using a manipulation that reduces or eliminates the TI percept) or in a between-group design (that compares people with and without TI). The second prediction cannot be satisfactorily tested using either paradigm because current analysis methods for spatial localisation of EEG and MEG data are too imprecise.

We are aware of two single-case reports published in the literature that in part in support of this model. Llinas et al. (1999) have collected spontaneous MEG data from a group of patients (Parkinson’s disease, neurological pain and major depression) including one person with TI. They report a higher power ratio between the 5–10 Hz band and the 10–15 Hz band for patients, compared to healthy controls. In another study, Llinas et al. (2005) explored the effect of TI masking in a single TI participant by recording 5 min of spontaneous activity in silence and a further 5 min when the TI was masked by noise. A source-based analysis focused on the change in the power spectra across these two epochs at a site in the left auditory cortex. Theta (4–8 Hz) activity decreased when TI was masked compared to when it was heard. Since no data were shown for frequencies above 20 Hz, it was not possible to establish whether there were any differences in the gamma band. Moreover, the authors do not present results of statistical analysis performed on this data. A group-based study (*N* = 10) is consistent with TI-related effects on low-frequency activity in which a normalised measure of delta band activity (1.3–4 Hz) was lower during periods of residual inhibition following a tone stimulus, compared to control periods when a tone stimulus failed to evoke residual inhibition (Kahlbrock and Weisz 2008).

A number of MEG/EEG studies have compared oscillatory activity in TI participants and normal-hearing controls using sensor-based analyses (Weisz et al. 2005; Ashton et al. 2007; Weisz et al. 2007b). For example, Weisz et al. (2005) observed an increase in MEG oscillatory power in the delta (1.5–4 Hz) frequency band and a simultaneous reduction in the alpha (8–13 Hz) frequency band in people with TI and hearing loss compared to normal hearing controls with no TI. A subsequent study by the same group confirmed the increase in delta (1–3 Hz) activity and decrease in alpha (8–12 Hz) for a similar between-group comparison (Weisz et al. 2007b). Recently, Zeng et al. (2011) have reported an interesting case study of TI reduction following a brief burst of electrical stimulation in a cochlear-implant user. The time-frequency representation of the brain oscillations at a frontal site (between 200 and 600 ms after stimulation) revealed a decrease in alpha (7–12 Hz) activity when TI was present relative to a state of TI suppression. In the healthy auditory system, alpha activity may be the inhibitory mechanism that maintains the necessary balance between excitation and inhibition. In TI, reduced alpha activity reflects loss of inhibition, which in turn results in spontaneous synchronisation and enhanced gamma activity. These ideas have been fully explored in a recent review of auditory alpha activity by Weisz et al. (2011).

Regarding gamma activity in TI, Weisz et al. (2007b) showed abnormally high levels of oscillatory power in the gamma band for the TI group, particularly in the 50–60 Hz range. EEG has also been used to demonstrate an increase in gamma activity (40–80 Hz) that was maximal around 60 Hz over the temporal cortex and was putatively associated with TI (Ashton et al. 2007). Groups were not matched for hearing loss because the hearing status of the ‘no TI’ controls was unknown. Some of the reported effects do not appear to withstand replication. For example, Kahlbrock and Weisz (2008) examined oscillatory activity in the gamma band, but did not find it to be affected by residual inhibition. Ashton et al. (2007) found increase in the gamma band for TI compared to controls, but no evidence of abnormal activity in any of the low-frequency bands.

In our recent review of the underlying mechanisms for TI, we highlighted numerous challenges in the use of human neuroimaging to assess some of the potential mechanisms (Adjamian et al. 2009). One issue concerns the difficulty in translating from microscopic neural events, recorded in animals with TI, to macroscopic patterns of brain activity recorded in humans with TI. However, the second issue is perhaps more tractable, and this concerns the rather poor control over co-morbid factors. In our review, we therefore recommended that the characteristics of participants forming a ‘TI group’ are as closely matched as possible, in terms of aetiology, duration, severity and laterality of TI, age, audiometric profile and other relevant co-morbid factors. None of the between-subject designs described above has matched their control group for degree of hearing loss. As a consequence, they cannot come to any unequivocal conclusions about whether oscillatory activity relates specifically to TI or to hearing loss. In our view, they do not therefore constitute a strong test of the thalamocortical dysrhythmia model for TI.

In the present study, we systematically assess the predictions of the thalamocortical dysrhythmia model (Llinas et al. 2005) using carefully matched cohorts of participants and then measure the changes in neural activity when the TI is modulated by a noise masker. One of the main questions is whether the changes in oscillatory activity within different frequency bands are associated with TI or hearing loss. In accordance with the thalamocortical dysrhythmia model, we expect elevated slow-wave (delta/theta) activity in TI patients with hearing loss compared to normal-hearing controls with no TI. If the slow-wave activity is a consequence of disrupted thalamocortical coherence due to deafferentation, we also expect elevated delta and/or theta activity in people with hearing loss, even if no TI is reported. However, if these low-frequency oscillations are specifically related to the TI condition, we would expect a reduction in their magnitude in participants who experience reduction in their TI with masking. The thalamocortical dysrhythmia model suggests that gamma-band activity is the correlate of the TI percept. We therefore expect to observe abnormally elevated gamma activity in people with TI regardless of hearing loss, and this should be reduced when TI is alleviated by masking. The thalamocortical dysrhythmia model makes no specific predictions about the ‘no tinnitus with hearing loss’ group. We might expect that the deafferentation would again be sufficient to drive increases in the low-frequency oscillations, perhaps without the concomitant changes in gamma that seem to underlie the positive clinical symptomatology.

## Methods

### Participants

Participants with TI and/or hearing loss were recruited from the Nottingham Ear, Nose and Throat (ENT) clinic, Nottingham Audiology Services and NIHR Nottingham Hearing Biomedical Research Unit. Participants with no TI or hearing loss were recruited from the general population. All participants in the TI groups had chronic subjective TI (for at least six months prior to recruitment). Individuals with pulsatile TI, Ménière’s disease, stapedectomy, and neurological disorders were excluded. All of the 52 participants were right handed as assessed by the Edinburgh handedness inventory (Oldfield 1971). Ethical approval was obtained from the Nottingham National Research Ethics Service (National Health Service), and all participants gave informed consent prior to their enrolment.

Participants formed four groups: (1) TI with hearing loss (*N* = 22); (2) TI with clinically normal hearing (*N* = 8); (3) no TI with hearing loss (*N* = 8); and (4) no TI with clinically normal hearing (*N* = 14). TI groups were matched for age, hearing loss (pure-tone average between 0.25 and 8 kHz), TI severity, and hyperacusis (Table 1). We had initially planned to recruit 16 participants in each of our experimental groups based on previously published guidelines for imaging studies (Friston et al. 1999). However, as we explain in ‘Discussion’, this proved more difficult for various reasons (e.g. exclusion criteria, necessity for matching across groups).

**TABLE 1:** Characteristics of the four groups of participants

		TI with hearing loss	TI with normal hearing	No TI with hearing loss	No TI with normal hearing
Gender	Male	14	2	4	6
	Female	8	6	4	8
Age	Mean (SD)	53.9 (9.9)	41.0 (14.8)	64.3 (8.8)	42.3 (14.4)

Pure-tone average (0.25–8 kHz)	Mean left (SD)	31.0 (17.9)	8.1 (6.8)	31.1 (15.2)	6.5 (6.2)
	Mean right (SD)	25.0 (16.4)	5.6 (6.2)	28.4 (17.9)	5.2 (5.8)

Pure-tone average (0.25–4 kHz)	Mean left (SD)	25.9 (17.8)	7.2 (7.1)	22.9 (15.4)	4.8 (6.1)
	Mean right (SD)	20.1 (15.7)	5.3 (6.9)	21.0 (18.5)	4.1 (5.6)

Tinnitus handicap (THI)	Mean (SD)	37 (22.5)	49 (20.5)	N/A	N/A

Hyperacusis	Score	36	31	0	0

TI quality	Tonal	13	7	N/A	N/A
	Hissing	4	0	N/A	N/A
	Ringing	5	1	N/A	N/A
TI laterality	Right	5	1	N/A	N/A
	Left	7	3	N/A	N/A
	Bilateral	10	4	N/A	N/A
Masking MEG	Yes	14	7	N/A	N/A
	No	8	1	N/A	N/A
TI duration (years)	Mean (SD)	12.6 (12.7)	5.2 (8.1)	N/A	N/A

### Audiometric characteristics

The degree of hearing loss was defined using clinical criteria and pure-tone audiometry was conducted for frequencies between 0.25 and 12 kHz. Normal hearing was defined as thresholds ≤20 dB hearing level (HL) at standard audiometric frequencies from 0.25 to 8 kHz. Given the age of our participants, clinically normal hearing does not necessarily mean normal auditory processing in the periphery, or absence of hearing loss and deafferentation at higher frequencies. Cochlear status cannot be verified without psychophysical measurement, which is beyond the scope of the current study. Nevertheless, the important factor in the present study is that we attempted to match participants in audiometric profile, which is an improvement on many previously reported TI studies.

**FIG. 1:**
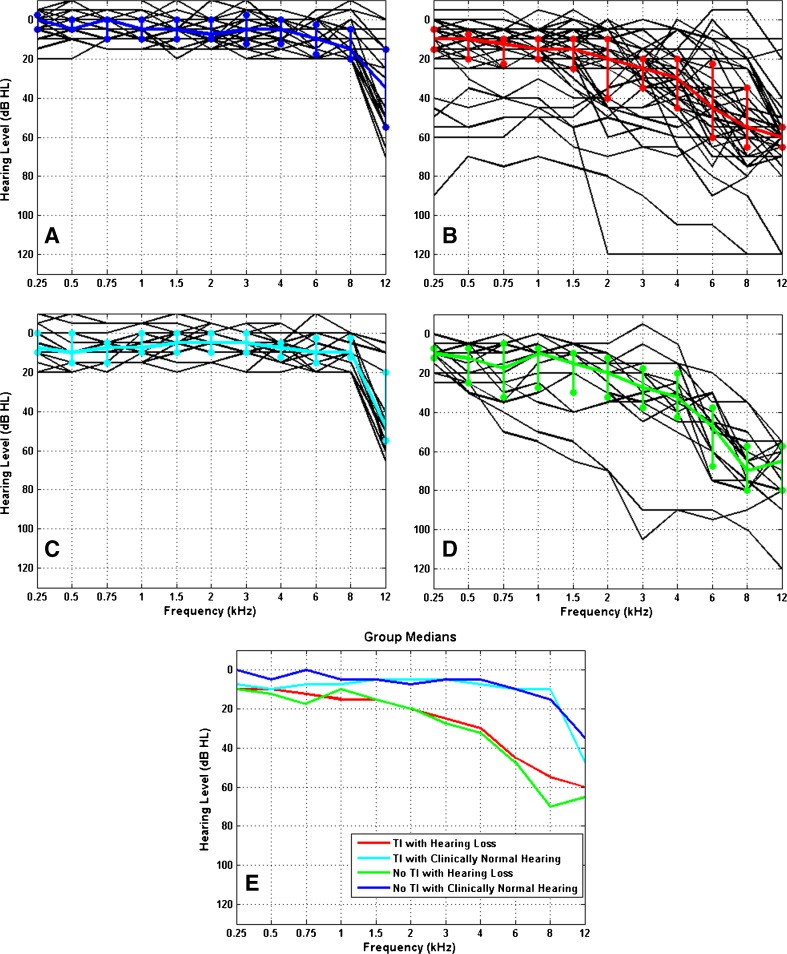
Individual (*black lines*) and median (*coloured lines*) audiograms for all ears in each group (**A–D**): no TI with clinically normal hearing (*blue*, *n* = 28), TI with hearing loss (*red*, *n* = 44), TI with clinically normal hearing (*cyan*, *n* = 16), no TI with hearing loss (*green*, *n* = 12). The variability across participants in each group is indicated using the 25 and 75 % interquartile ranges. **E** Superposition of the medians from **A–D**. Note the close matching for the groups with and without hearing loss.

The individual as well as median hearing thresholds for the four groups are shown in Figure 1A–D, which illustrates the thresholds for all pairs of ears in each group. The variability in hearing thresholds for each group is also depicted using the 25 and 75 % interquartile ranges. Figure 1E shows the group medians illustrating the good matches between the two clinically normal and the two hearing loss groups.

### Tinnitus characteristics

For participants reporting TI, severity of the symptoms was assessed by the Tinnitus Handicap Inventory, Newman et al. (1996). TI handicap was classified as none (0–16), mild (18–36), moderate (38–56) or severe (58–100) (McCombe et al. 2001). According to these categories, 14 participants reported mild TI, 8 moderate and a further 8 severe TI. Hypersensitivity to sounds was assessed with a questionnaire where a score of >28 is indicative of hyperacusis (Khalfa et al. 2002). According to this criterion, two participants were classified as hyperacustic (one in each TI group).

Tinnitus Tester software (Roberts et al. 2006, 2008) was used to measure TI laterality, loudness, dominant pitch and quality (cf. Sereda et al. 2011). With this procedure, participants make a loudness rating on a visual analogue scale (0–10). Pitch ratings of TI are made by comparison to a range of pure tones (0.5–12 kHz): this procedure is repeated three times, and the average likeness rating (0–100) is obtained. Another test in the Tinnitus Tester classifies TI quality by comparison with reference sounds that are pure tones (tonal TI), narrow band noise +/− 5 % of centre frequency (ringing TI) or narrow band noise +/− 15 % of centre frequency (hissing TI).

### Brain imaging methods (MEG and anatomical MRI)

MEG data were acquired with participants in supine position, in a magnetically shielded room using a whole-head CTF system (VSM MedTech, Port Coquitlam, Canada) with 275 radial gradiometers over the scalp at a sampling rate of 600 Hz. An additional 29 reference gradiometers and magnetometers were recorded for ambient noise cancellation (Vrba and Robinson 2001). Participants were instructed to fixate on a spot in the room and were told of the requirement to remain attentive. Using a camera inside the room, participants were observed and alerted by the experimenter via an intercom if sleep was suspected. These trials were noted and excluded from data analysis.

Three electromagnetic head coils were attached to the nasion, left and right preauriculars in order to localise the head relative to the MEG sensors before and after the recording session. For localisation of activation, MEG data were co-registered to individual MRI anatomical scans that were obtained using either a Philips 3 or 1.5 T scanner, depending on scanner availability. Minor differences in image quality and contrast across the two systems have negligible effect on the source reconstruction analysis. For each participant, between-modality co-registration was performed by obtaining a digitised shape of the head to match an extracted MRI scalp surface (Adjamian et al. 2004a).

### Stimuli and paradigm

**FIG. 2:**
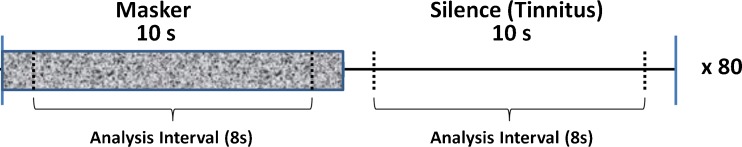
The sequence of MEG data acquisition: each trial of 20 s consisted of 10 s of the masker sound followed by 10 s of silence in which participants with TI were able to hear their TI. Eighty repetitions of the trial were presented.

The noise masker comprised frequencies of 0.1–12 kHz and was 10 s in duration (including rise and fall ramps). The masker was presented in the MEG system using Presentation (Neurobehavioural Systems) via a TDT System III (Tucker-Davis Technology) and small transducers (Etymotic Research—ER2). Each transducer was placed in specially designed mu-metal casing to protect the MEG sensors from magnetic interference, and the sounds were delivered to the participants via short plastic tubes. The sound level was set to 50 dB SPL for all participants. The recording session comprised 80 trials of 20 s duration as shown in Figure 2 (10 s masker and 10 s silence). We preferred this passive listening paradigm rather than one with a task involved. While active listening tends to cause arousal and attention to the TI, passive listening tends to soothe and facilitate masking (Sweetow and Henderson-Sabes 2010), providing the ideal conditions to observe changes in the ongoing oscillatory activity of the brain.

Following the recording, participants indicated their TI loudness on a visual analogue scale (0–10), both during the masker and the silent epochs. The amount of attenuation of the TI was calculated by subtracting the score during masking from the score during silent periods. A positive score indicates some degree of attenuation, while a negative score indicates an increase in loudness. For the MEG analysis, any degree of reported TI attenuation was regarded as ‘masked’ (even if it was only one unit on the visual analogue scale).

### MEG sensor-based analysis using power spectra

For both sensor- and source-based analyses, the first step involved removal of signal artefacts. The MEG data were first visually inspected and trials with obvious artefacts (channel resetting or strong muscle activity) were rejected. The remaining data were corrected for eyeblinks and heartbeat-related artefacts using an independent component analysis approach implemented in the Fieldtrip toolbox (http://fieldtrip.fcdonders.nl/start) (Oostenveld et al. 2011) that utilises the EEGLab toolbox for this analysis (Delorme and Makeig 2004). The CTF MEG system consists of first-order axial gradiometer sensors, which measure the radial component of the magnetic field orthogonal to the scalp surface. The interpretation of sensor data thus becomes more difficult given the ambiguities in resolving probable sources. To mitigate this problem for the analysis of sensor data, we calculated the first spatial derivative by transforming the data to a planar configuration using the Fieldtrip toolbox. The amplitude of the planar gradient is then computed for each sensor by combining the horizontal and vertical components of the planar gradient. The power spectral density for the masker and silence epochs was obtained for each dataset in the 1–80 Hz band using a Hanning taper that offers acceptable smoothing of frequency data to analyse the spectrum of the segment of interest (Mitra and Pesaran 1999). Based on these results we investigated spatio-spectral differences between groups. For each subject, power at each sensor was calculated in frequency bands of interest and averaged over all trials. Based on the average power, we computed two-sample independent *t*-statistics for all channels using a permutation test with multiple comparison applying the Bonferroni correction. It must be noted here that the beamformer technique and other source analysis techniques, in general, are more precise measures of spatial and temporal activity. Nevertheless, sensor analysis of power spectra was performed on the silence epochs mainly for illustrative purpose and comparison of the results with previously published work of other groups who have reported results based on sensor space analysis (e.g. Weisz et al. 2005; Ashton et al. 2007).

For both sensor and source analyses, we were interested in spontaneous activity during silence epochs and the effect of masking on the ongoing spontaneous brain activity. Therefore, we excluded the contribution of large evoked responses occurring at the onset and offset of each masker signal by removing the first and last second of each masker and silent epoch (i.e. the analysis window was 1–9 s instead of 0–10 s).

### MEG source analysis using beamformer region of interest

The preceding sensor-space analyses consider activity across the whole head and do not necessarily reflect the activity of the underlying cortex, but rather the summed activity from multiple sources across variable distances. Furthermore, they are susceptible to various unrelated magnetic fields, which can distort the neural activity of interest. Source-space analyses are preferable because they limit the analysis to activity that originates within auditory cortex. To estimate the source of focal oscillatory activity in various frequency bands, a spatial filtering technique using the adaptive beamforming method, details of which are given elsewhere (Robinson and Vrba 1998; Hillebrand and Barnes 2005). A three-dimensional image of source activity is generated using un-averaged data by plotting source power as a function of noise variance derived from the covariance of all the data. Normalisation by the noise variance produces images of pseudo-statistics dependent upon source activity and independent of depth. This image is then overlaid on the co-registered anatomical brain scan for each participant.

To perform a direct test of the predictions made by the thalamocortical dysrhythmia model (Llinas et al. 1999, 2005), each dataset was first bandpass filtered in the frequency bands of interest: delta (1–4 Hz), theta (4–8 Hz), alpha (8–13 Hz), beta (13–20 Hz) and gamma bands of 10 Hz wide bins (25–35 Hz, 35–45 Hz, 45–55 Hz, 55–65 Hz and 65–75 Hz). The beamformer was used to identify oscillatory power in the entire brain volume in the masker and silent epochs separately. Power is presented as pseudo-*z* (p*Z*) values with each *z* being a normalisation of the estimated signal to the estimated sensor noise (*z* = signal/noise). Thus, single-state images of the ratio of source power to noise variance were generated for the masker and silence epochs, which resemble the *z*-deviate of cortical activity. Volumetric images were generated in 0.5 cm voxels within each frequency band, for the masker and silent epochs separately.

**FIG. 3:**
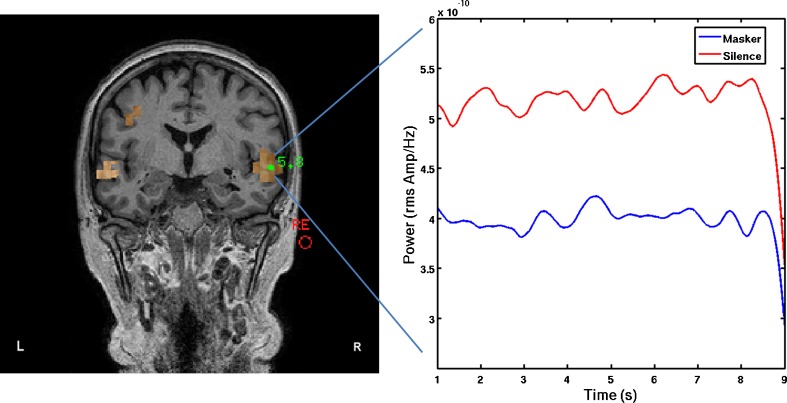
**A** Beamformer pseudo-Z image of auditory cortical activation in the delta (1–4 Hz) band during the silence condition for one participant (TI with hearing loss). **B** For the right hemisphere peak voxel depicted in **A**, the time course of the normalized power in the delta band (1–4 Hz) is plotted across the masking and silence epochs.

Further analysis of changes in oscillatory activity was performed on the *peak* location in auditory cortex, irrespective of hemisphere. Here, ‘auditory cortex’ was defined as the superior and middle temporal gyri and sulci, and the ‘peak’ was identified from the single-state image for the silence epochs, which was a different location in each of the frequency bands of interest. In nine participants, beamformer analysis of the gamma-band power failed to identify any peak within auditory cortex, and hence, these data were deemed insufficiently reliable for further analysis. For the remaining frequency band analyses, there was no apparent relationship between the side of the TI sensation and the hemisphere containing the peak. The peak coordinate was then used to estimate power as a function of time (over the 8 s window). Time–frequency wavelet maps were measured for each voxel virtual electrode to obtain an estimate of power over time in each frequency band. The same peak location was then used to compute time frequency maps for the masker epochs in order to observe oscillatory changes at the same location due to the presentation of the masker (see Fig. 3). The time course of activity in each frequency band was then plotted for the silence and masker conditions in each frequency band. An estimate of difference in normalised power was obtained by averaging across the time points, which was then used for statistical testing.

## Results

### MEG sensor analysis using power spectra

**FIG. 4:**
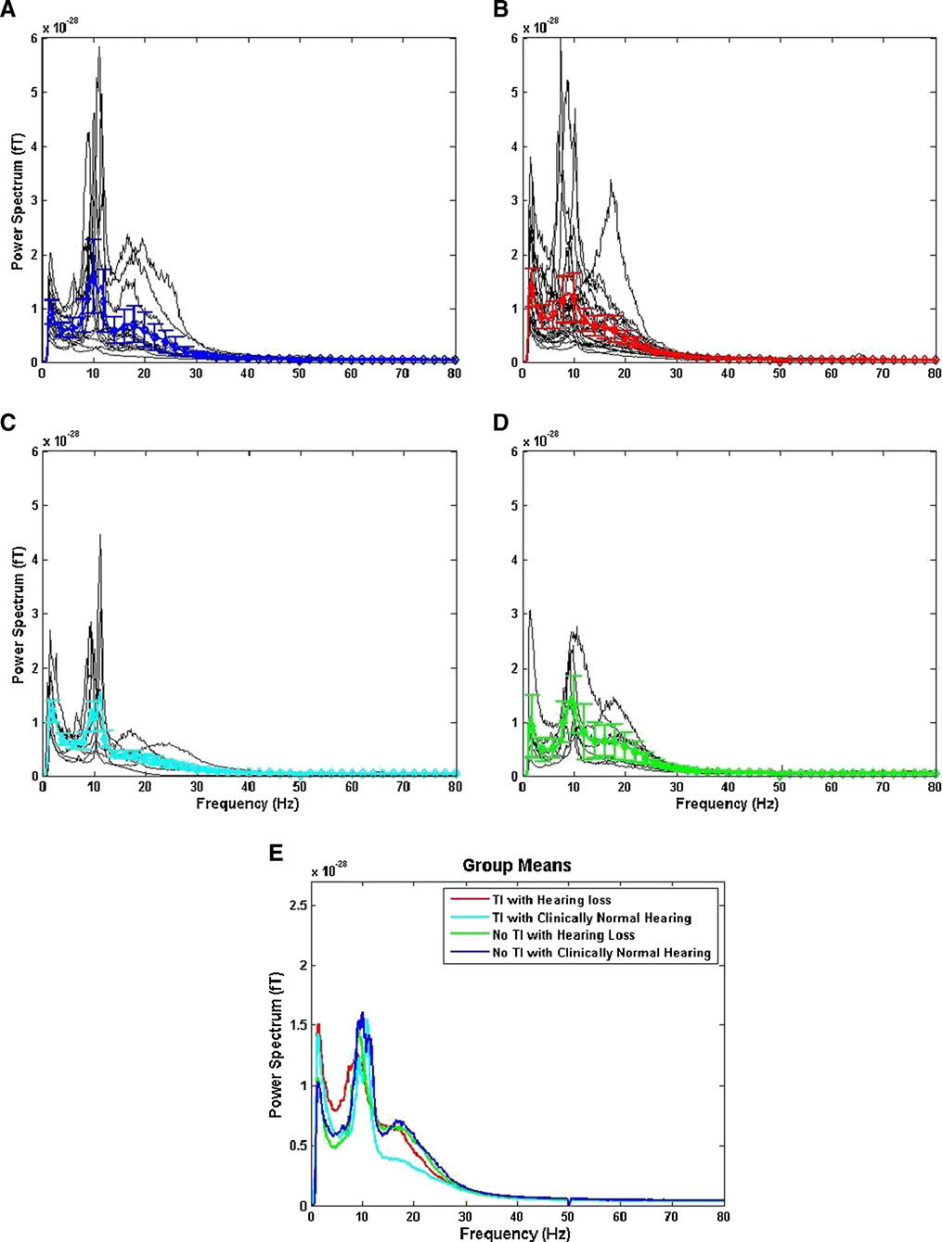
Multi-taper power spectra (**A–D**): individual (*black*) and mean spectra (±SEM) for no TI with clinically normal hearing (*blue*, *n* = 14), TI with hearing loss (*red*, *n* = 22), TI with clinically normal hearing (*cyan*, *n* = 8) and no TI with hearing loss (*green*, *n* = 6). **E** Superposition of means from **A–D**. Note that no differences exist between individuals or groups at higher frequencies.

One of the main questions is whether the changes in oscillatory activity within different frequency bands are associated with TI or hearing loss. In accordance with the thalamocortical dysrhythmia model, we expected elevated slow-wave activity (in the delta and/or theta band) in ‘TI with hearing loss’ participants compared to the ‘no TI, normal hearing’ controls. Figure 4 shows the individual and group-averaged results of the power spectral density analysis of the MEG sensor data for the silence period. The average (Fig. 4E) shows enhanced delta activity for the ‘TI with hearing loss’ and ‘TI with normal hearing’ groups compared to other groups. Furthermore, in TI with hearing loss there was an increase in theta (∼7 Hz) and a subsequent reduction in alpha (∼10 Hz) band activity compared to other groups. To assess whether these mean differences were statistically significant, we obtained the average of the spectral time points for each participant across all channels and performed independent *t* tests using the group mean spectra. Confirming previous published results, there was a significant difference in delta band (1–4 Hz) power between the ‘TI with hearing loss’ and ‘no TI with normal hearing’ groups (t(34) = 2.33, *p* = 0.026). However, this was not significant in either the theta band (4–8 Hz) [*t*(34) = 1.66, *p* = 0.105], or in the alpha band (8–13 Hz) [*t*(34) = −0.79, *p* = 0.434]. It is interesting to note that *t*-statistic comparisons between the ‘TI with normal hearing’ and the ‘no TI with normal hearing’ groups and between the ‘TI with hearing loss’ and the ‘no TI with normal hearing’ did not reach significance (*p* > 0.05). This pattern of null results is not confirmatory, but is at least consistent with the view that a combination of TI and hearing loss is necessary for the observed increase in delta-band activity. There was no difference between the groups in the gamma band for frequencies up to 80 Hz (*p* > 0.05).

**FIG. 5:**
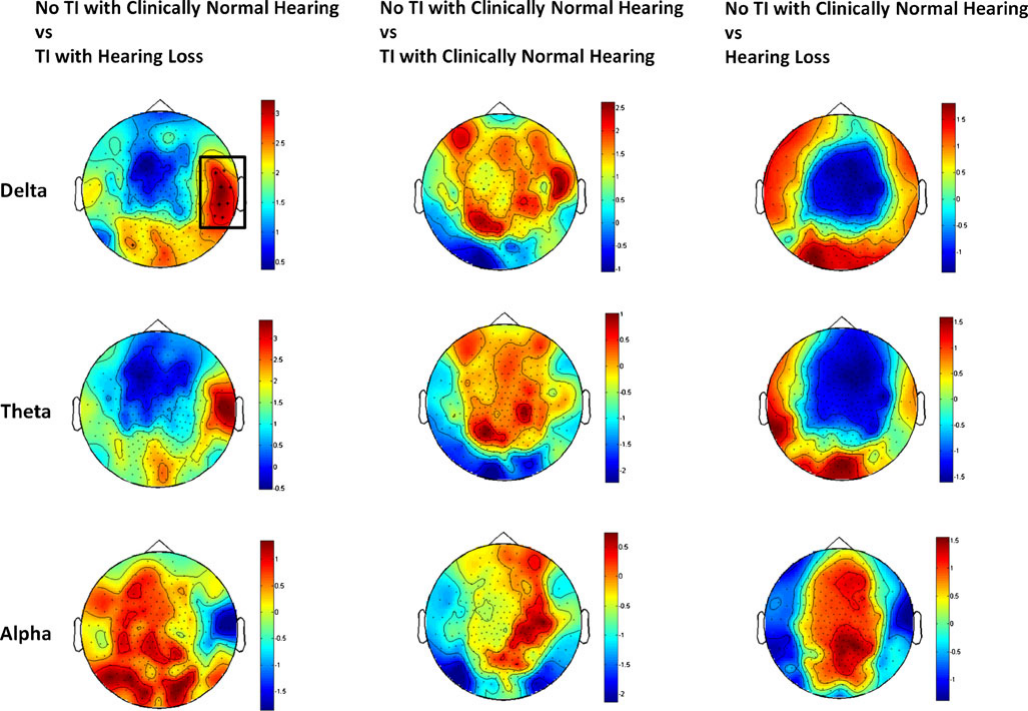
Representation of spatio-spectral differences between groups. The plot shows *t*-statistical differences at the sensor level for group of participants for the delta, theta and alpha bands. The statistical test performed is a permutation test, using the Bonferroni correction (alpha = 0.001). The *colour bar* indicates the *t*-statistic. The only differences was between TI with hearing loss and the no TI with clinically normal hearing groups in the delta band for channels above the right auditory cortex.

The results of spatio-spectral differences between groups in the delta, theta and alpha frequency bands are shown in Figure 5, in which channels with significant between-group differences are highlighted. There is a significant increase in delta activity for channels corresponding to the right auditory cortex between the ‘TI with hearing loss’ and the ‘no TI with clinically normal hearing’ groups. No significant differences were found in other frequency bands between groups as also indicated from the spectral analysis shown in Figure 4.

**FIG. 6:**
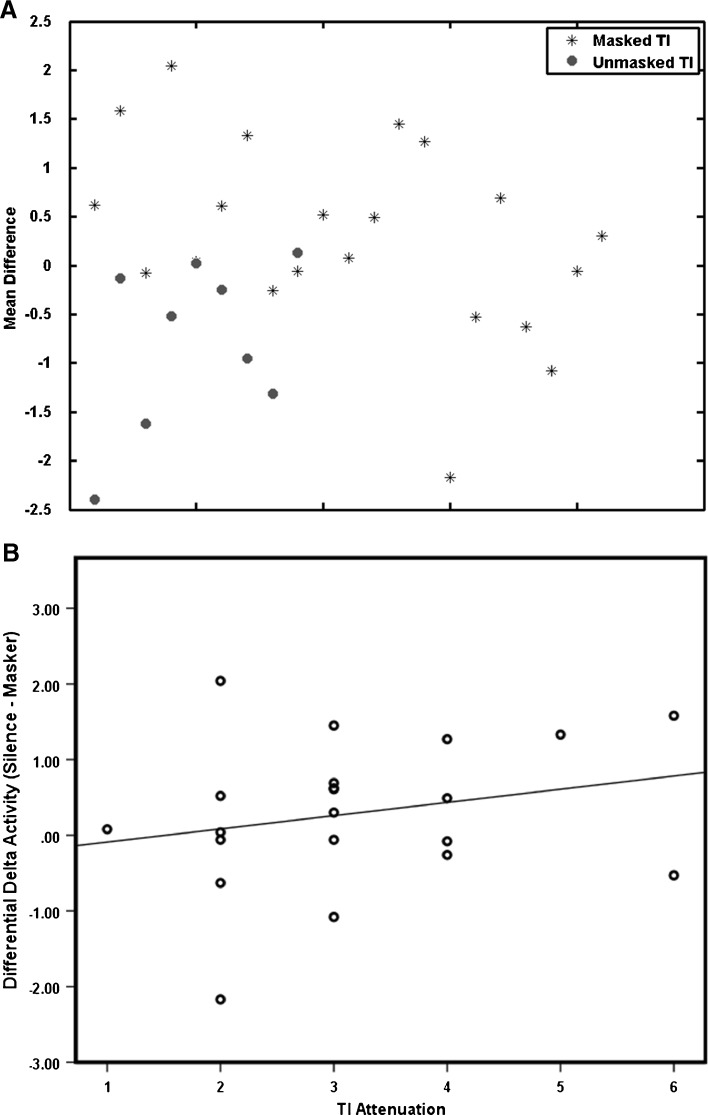
Estimates of TI masking: **A** mean difference between masker and silence in the delta band for participants who experienced TI reduction through masking (*blue*; *N* = 21) and those who did not (*red*; *N* = 9); **B** correlation between amount of reduction in delta with TI reduction through masking and the level of TI attenuation as reported by each individual.

To further investigate the observation made by Weisz et al. (2005, 2007b) that the enhanced delta activity might be related to the TI percept, we reasoned that the magnitude of delta power in both TI groups might subsequently be reduced by masking in participants who experienced its effect, but not in participants in whom the TI percept remained unchanged. Of the 30 participants who experienced TI (irrespective of hearing loss), 21 reported their TI to be attenuated by the masker (3 with total and 18 with partial masking) during the MEG experiment, while the remaining 9 reported no change in TI percept. Of the 21 who experienced masking, 7 had clinically normal hearing. All participants indicated that their TI returned immediately after the 10 s masker. To assess the association between TI percept and oscillatory brain activity, the data were analysed in the following steps. Delta band power was calculated across the 8 s window (1–9 s) of the silence and masker epochs, and data points were then averaged over time to provide a single value representing the mean delta band power in each separate epoch. The ‘masker’ value was subtracted from the ‘silence’ value such that a positive result indicated a reduction in delta band activity during the masker and a negative value indicated an increase. Figure 6A illustrates the result of this analysis. Of the 21 participants with TI who experienced attenuation of their TI during the masking condition, 13 showed a concomitant reduction in delta-band activity (including 3 with normal hearing), while from the 9 who did not experience TI masking only 2 showed reduction in delta activity. The differential delta activity was significantly different between the two groups [*t*(28) = 2.87, *p* = 0.007]. However, further analysis using a partial correlation that accounted for the degree of hearing loss, failed to reveal a significant relationship between TI attenuation and the difference in delta-band activity (Fig. 6B) (*r* = 0.235, *n* = 21, *p* = 0.319).

### MEG source analysis using beamformer region of interest

The specific predictions made from the thalamocortical dysrhythmia model were assessed using planned pair-wise comparisons. First, we expected elevated slow-wave (delta and theta) activity in the ‘TI with hearing loss’ group compared to the ‘no TI with clinically normal hearing’ controls. An independent *t* test revealed this to be significant for both delta [*t*(34) = 3.07, *p* = 0.004] and for theta [*t*(34) = 2.34, *p* = 0.025].

**FIG. 7:**
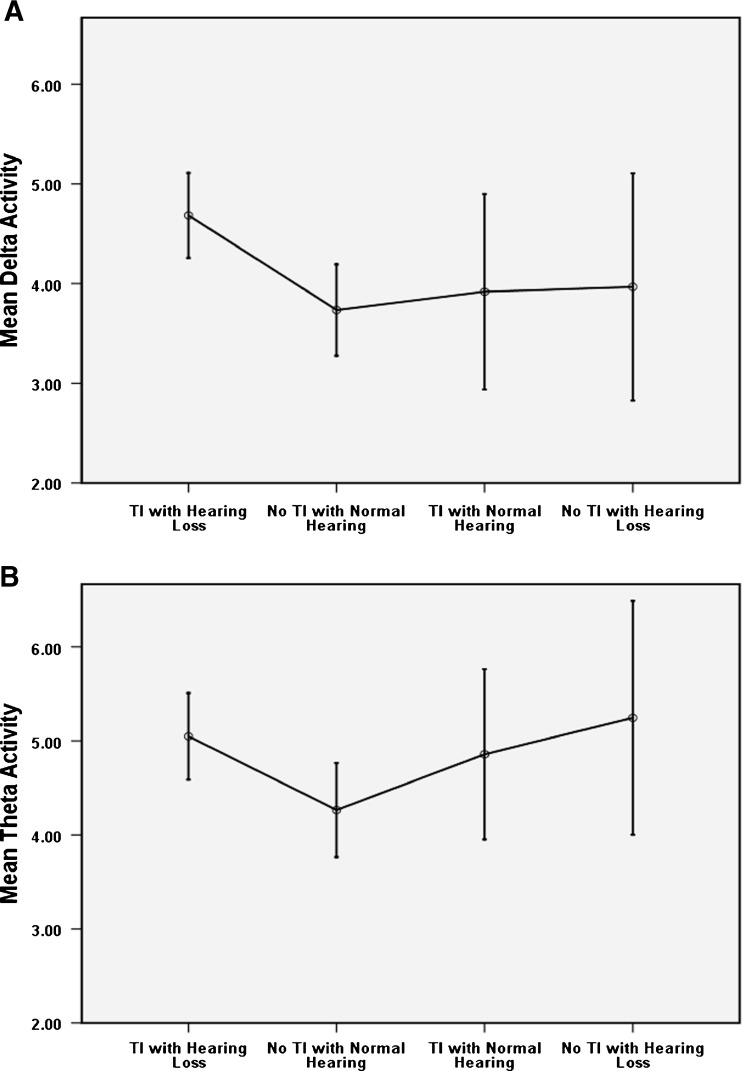
The means (95 % interval) of groups for the delta (**A**) and theta (**B**) activity showing overall enhancement for ‘TI with hearing loss’ compared to the ‘no TI with normal hearing’ group.

If the slow-wave activity were a consequence of disrupted thalamocortical coherence due to deafferentation per se, then we would expect elevated slow-wave activity in people with hearing loss, even if no TI was reported. However, this was not supported by the data. Collapsing across participants with hearing loss, this contrast did not reach significance for delta [*t*(42) = 2.30, *p* = 0.27]. Figure 7 shows the mean and 95 % confidence intervals for the delta and theta activity across groups. This pattern of findings again is consistent with our interpretation of the sensor-space analysis that a combination of TI *and* hearing loss is needed to drive the abnormal increases in slow-wave activity. The thalamocortical dysryhthmia model also predicts that gamma activity should be enhanced as a correlate of the TI sensation. The independent *t* test did not reveal a significant difference between TI with hearing loss and no TI with normal hearing groups [*t*(42) = 0.746, *p* = 0.462].

We also performed exploratory analysis between the groups using a one-way ANOVA to assess for possible effects in other frequency bands between all groups. The results revealed no significant differences in other groups and other frequency bands.

### TI masking estimate using the beamformer

**FIG. 8:**
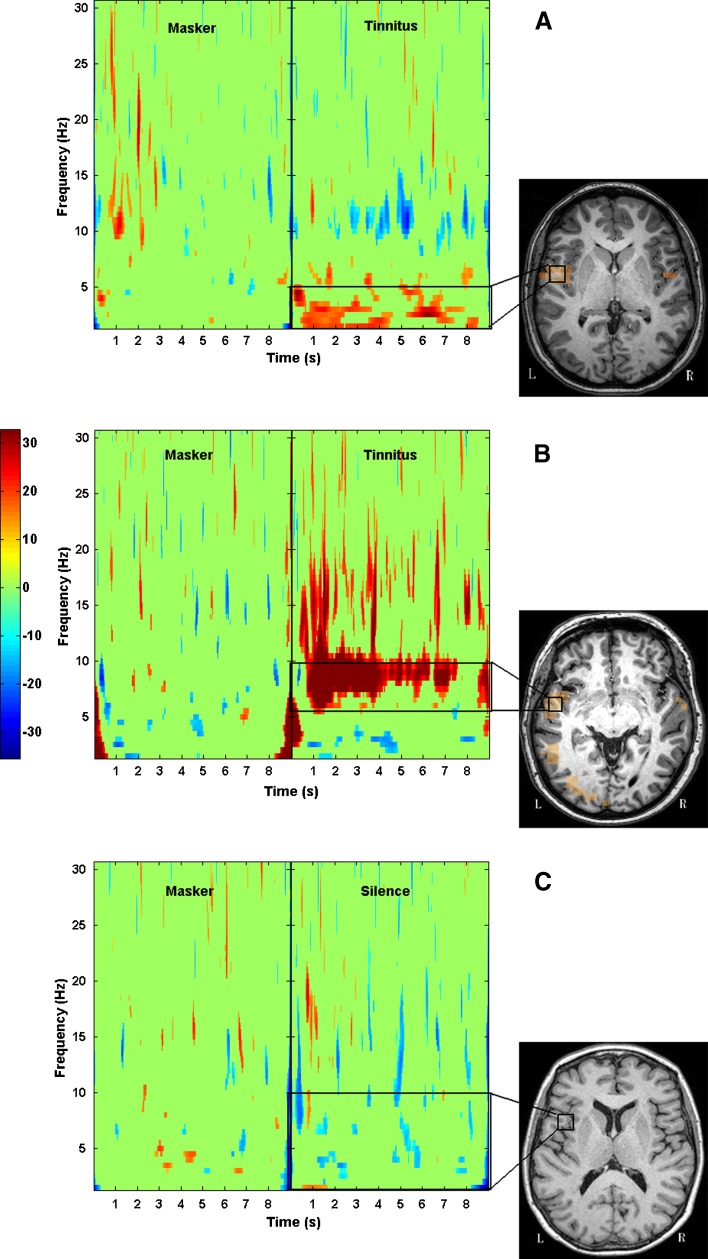
Sample wavelet time–frequency spectrogram from two participants with TI and hearing loss who experienced reduction of their TI with masking, and a participant with no TI and clinically normal hearing. Peak locations were identified in the delta (**A**) and theta (**B**) bands using beamformer in the silence period when TI was present. The virtual electrode time-series corresponding to these locations were extracted for time-frequency analysis using a bootstrap technique. Power fluctuations in the tinnitus epochs are calculated relative to the masker epochs, which are treated as baseline, with values averaged to zero. **A** Enhanced delta activity appearing with the onset of TI percept. **B** Onset of theta activity following the masker in another TI patient. Similarly, **C** shows the same analysis in a participant with no TI and clinically normal hearing. The virtual electrode was calculated in the delta band (1–5 Hz) in this case showing no significant increase/decrease in power for the silent period.

As shown in our sensor space analysis, the slow-wave activity is specifically associated with TI, and such activity in auditory cortex is reduced during periods when the TI is attenuated or masked. Here, we conduct an analysis in source space of the change in delta wave activity due to the masking noise. Loudness estimates during the experiment confirmed that 21 participants experienced some degree of TI attenuation when listening to the masker. A decrease in oscillatory power in the delta band was measured for 15 of those 21 participants. A paired *t* test on the means of the time courses for the masker and silence conditions revealed this difference to be significant across the whole group [*t*(20) = −3.50, *p* = 0.002]. Eleven participants also showed a reduction in oscillatory power in the theta band as a result of TI masking, but this did not reach significance [*t*(20) = −1.74, *p* = 0.098] for the group. For each participant, we extracted the time course of activity at the peak location corresponding to the pseudo-z peak (the virtual electrode) and performed a time–frequency analysis using a bootstrap technique (Graimann et al. 2002). In this analysis, the masker condition is regarded as the baseline, and the average power in this condition is taken as reference. Changes in oscillatory power during silence (when TI is perceived) are computed relative to this baseline. Figure 8 shows spectrograms for two participants showing higher levels of delta and theta activity during TI compared to the epoch when the masker was present.

## Discussion

To our knowledge, this is the first study to investigate spectral changes associated with masking TI in human auditory cortex, within the context of a between-subject design that controlled for hearing loss, as well as for the presence of TI. We assessed different frequency bands of the cortical oscillatory activity in TI participants with and without hearing loss. Source- and sensor-space analysis of the changes in oscillatory power revealed comparable results, which were partly in agreement with the thalamocortical dysrhythmia model (Llinas et al. 2005). Sensor-space analysis revealed significant increases in delta-band activity in people with TI and hearing loss compared to the ‘no TI with normal hearing’ group, and this effect was localised to auditory cortex using beamformer analysis. TI participants with no hearing loss and ‘no TI, normal hearing’ controls did not significantly differ in their slow-wave or gamma-band activity. We further found that delta activity reduced in TI participants who experienced masking compared to those who did not experience its effect, although the amount of TI attenuation did not correlate with the amount of delta reduction. Our findings demonstrate that slow wave activity may be considered the neurological signature of conscious TI perception.

### Slow-wave activity (delta/theta)

Slow wave oscillatory activity (<8 Hz) appear to be a prominent characteristic of TI. Weisz et al. (2007b) propose a model similar to thalamocortical dysrhythmia, according to which synchronous neural activity due to deafferentation underlie the abnormalities in TI which can be observed as changes in oscillatory activity with MEG or EEG. In short, lesions to inner ear structures lead to a suppression of excitatory neural activity and the activity of inhibitory neurons. Delta activity is the slowing of activity mediated by signal deprivation, whereas gamma activity reflects the synchronised firing of neurons mediating the conscious perception of TI. The difference with thalamocortical dysrhythmia model is that gamma activity, rather than being an ‘edge effect’, is due to the inhibition of alpha activity, which may be driven by thalamic afferents or may be a release of inhibition as spontaneous synchronisation of excitatory neurons in the auditory cortex. Previously, Weisz et al. (2005) reported abnormally increased delta activity (1.5–4 Hz) in temporal regions and a simultaneous decrease in alpha band (8–13 Hz) activity, which they attribute to disinhibition of normal brain rhythms. Kahlbrock and Weisz (2008) have made use of the effect of residual inhibition to assess cortical oscillatory activity in TI. They found that the reduction of TI percept during residual inhibition resulted in a concomitant reduction of delta band power. In accordance with this finding, we also found a reduction of delta activity with TI attenuation, even though the amount of perceptual attenuation and the decrease in cortical delta activity were not correlated. The absence of a significant correlation maybe explained by the fact that two entirely different datasets (subjective loudness rating with objective MEG activity) were correlated. Cortical slow-wave activity recorded with MEG/EEG may be the correlate of synchronised activity of large populations of neocortical neurons. Slow wave activity has been observed during various stages of sleep with several studies indicating that the thalamus might play an active role in shaping these oscillations (Steriade et al. 1993; Sirota and Buzsáki 2005). In the awake state, local slow wave activity in a circumscribed brain region has been reported under various psychopathological conditions, including dementia, schizophrenia and depression (Buchan et al. 1997; Fehr et al. 2001; Wienbruch et al. 2003). Slow wave activity has also been found in the vicinity of structural lesions such as cerebral infarct, tumours and epileptic foci (Tanaka et al. 1998; de Jongh et al. 2001; Ishibashi et al. 2002). It is therefore likely that they represent synchronised slowing of activity in large population of neurons with altered thalamic input due to neural deprivation. Abnormally enhanced cortical slow wave activity in the delta and theta range is one of the main predictions of the thalamocortical dysrhythmia model and supported by the results of our study.

### Gamma

It has been suggested that gamma activity in the auditory cortex is necessary for the conscious perception of the TI sound (Weisz et al. 2007b) and that it is its neurological signature (Weisz et al. 2005). Indeed, Llinas et al. (1999) suggested that gamma activity may be the clinical underpinning of phantom perceptions in general and the thalamocortical dysrhythmia model predicts an increased gamma activity as ‘edge effect’ in TI patients. In the EEG/MEG literature, reference to gamma activity spans broad spectrum of frequencies, most typically ranging from 25 to 80 Hz. In this study, we focused on the 40 Hz activity as this is the effect specifically implicated by Llinas et al. (1999, 2005). For this reason, the beamformer analysis frequency band (35–45 Hz) was selected to reflect this. Some previous TI studies report gamma activity between 50 and 60 Hz (Weisz et al. 2007a; Ashton et al. 2007) in sensor space; however, our sensor spectral power analysis did not reveal any differences between groups at these frequencies.

Gamma activity appears to be indicative of hyperactivation of central auditory structures and to play an important role in the generation of TI (Eggermont and Roberts 2004). A number of EEG/MEG studies have found gamma activity in TI. Weisz et al. (2007b) found 55 Hz gamma activity in temporal regions contralateral to the TI side and bilaterally in patients with bilateral TI and suggested that it is an index of the laterality of the TI. Van der Loo et al. (2009) found that auditory cortex gamma activity correlates with the perceived TI loudness. Specifically, they found that subjective measures of TI loudness correlated positively with current source densities in the gamma band in contralateral auditory regions. They concluded that gamma activity is not related to the conscious perception of TI per se, but to its perceived loudness and suggest that perhaps gamma activity reflects the intensity of the perceived sound. In another recent study, Ortmann et al. (2011) found increased gamma activity in auditory cortex in a group of rock band musicians directly following band practice and exposure to extreme sound intensities. All participants reported perceiving TI either bilaterally or to one side with an intensity of up to 30 dB SL after exposure. Temporary hearing loss was also present in both ears. However, no concurrent increase in slow wave or reduction of alpha activity was found and the results do not provide a causal link between the observed gamma activity and TI sensation in chronic TI sufferers. These earlier studies appear to contradict our findings here where we failed to find any significant change in gamma activity that correlates with TI. The role of fast oscillations in the brain is under much investigation and its functional significance is unclear, but is likely to be involved in several processes. For example, in the visual domain, it has been suggested that gamma activity represents perceptual binding (Tallon et al. 1995) as well as reflecting the fundamental features of stimuli (Adjamian et al. 2004b, 2008). In the somatosensory domain, Gross et al. (2007) demonstrated that painful stimuli induce gamma oscillations in the contralateral S1 cortex. These findings suggest that gamma activity plays a role in conscious perception of stimuli in various modalities. According to the thalamocortical dysrhythmia model, increased slow wave and gamma activity is mediated by deafferentation, implying that increased gamma activity is mediated by hearing loss. Thus, it would be expected that enhanced gamma activity is present whether or not TI exists, provided that there is some degree of deafferentation. However, our hearing loss groups also showed no enhanced gamma activity. This may be due to a number of factors, such as variability between TI sufferers or the complexity of the processes that underlie TI, or maybe a consequence of the analysis techniques employed. The effect of TI modulation on the strength of observed oscillatory activity requires further investigation.

### Participant recruitment

The recruitment of participants with TI and clinically normal hearing, and with no TI with hearing loss proved difficult. Despite recruiting participants over a continuous period of 24 months, we only successfully recruited eight suitable individuals for these particular groups. In the case of TI with clinically normal hearing, this proved difficult since most of our volunteers presented with co-morbid TI and hearing loss. In the case of participants with no TI and hearing loss, most of our initial volunteers tended to be from the elderly population, which was counter to our efforts to construct groups of participants with a similar average age. Others of suitable age had either co-morbid factors (e.g. stapedectomy) or such pronounced hearing loss that they were unable to hear the masking sound and had to be excluded from the MEG session. We thus acknowledge that this inequality in participant numbers may result in statistical analyses that are potentially underpowered. However, our results initially demonstrate that abnormal oscillatory activity occurs with TI regardless of hearing loss, and we would welcome further replications of the effects reported here.

### Classification of hearing loss

We would like to add a caveat regarding the classification of the ‘TI with clinically normal hearing’ group in this study, to which we also alluded in the introduction. One main purpose of this study was to carefully match groups on hearing level and assess changes in oscillatory power during silence when TI is perceived and when it is attenuated by the masker. However, it is possible that peripheral damage, especially at high frequencies, could be present, but not detected by routine audiometry. For example, Weisz et al. (2006) used the TEN test to assess inner hair cell damage in 11 TI patients with audiometrically normal thresholds and found cochlear dead regions in eight of the 11 patients. They suggest that TI without hearing loss does not necessarily indicate the absence of any deafferentation. Two further recent studies suggest that noise-induced damage may not be revealed by conventional threshold measurements. Kujawa and Liberman (2009) have shown that temporary hearing loss following intense noise exposure produces widespread loss of afferent nerve terminals and degeneration of the cochlear nerve. The recovery of audiometric thresholds to normal levels does not indicate the reversal of damage to inner ear structures, and this can lead to perceptual abnormalities such as TI and hyperacusis. Schaette and McAlpine (2011) suggest that TI patients with apparently normal audiograms may have ‘hidden hearing loss’, which is defined as damage to auditory nerve fibres, possibly due to exposure to loud noise, which is not detected by routine measurement of audiometric thresholds. Therefore, in this paper, ‘normal hearing’ refers to participants who had normal clinical audiograms, but we are aware that this classification does not suggest absence of deafferentation altogether.

Of the 21 TI participants who experienced TI masking, 7 had normal hearing, 3 of whom also showed an associated reduction in delta activity. This would indicate that TI masking could occur irrespective of hearing loss. The premise of the thalamocortical dysrhythmia model is that the abnormal thalamic dysrhythmia is primarily due to deafferentation, and thus, delta/theta activity should reflect this disruption in ‘communication’. The model proposed by Weisz et al. (2007a) also suggests that deafferentation is essential to the cascade of neural changes that occur. Although our patients had clinically normal hearing using the criteria defined earlier [thresholds ≤20 dB (HL) at standard audiometric frequencies from 0.25 to 8 kHz], and we only examined hearing thresholds up to 12 kHz, we cannot rule out hearing impairment at higher frequencies. Thus, it is likely that at least some of these participants had impaired hearing thresholds above 12 kHz. Only one of the eight participants in the TI with normal hearing group had thresholds >20 dB HL at 12 kHz. Hearing loss at higher frequencies can contribute to the manifestations of TI, and it may be argued that classification of these participants into a ‘normal hearing’ group is not warranted. However, since the same audiometric criteria were applied for the classification of the healthy control group, it is as likely that at least some of these also had impaired hearing at frequencies above 12 kHz. Although our MEG results clearly distinguish between participants with and without normal audiometric thresholds, we cannot rule out the conclusions by the aforementioned studies.

In the light of these findings, we suggest that TI is likely associated some degree of damage to the auditory system and that TI with ‘normal hearing’ based on audiometric thresholds may be a misnomer. We suggest that even small changes in threshold (<20 dB HL), at any audiometric frequencies, can have implications for the onset of TI. Regarding the cortical oscillatory activity associated with TI, our results indicate that slow-wave activity in TI is associated with TI itself rather than deafferentation alone. Whether TI without hearing loss exists is debatable, and it is more likely that all TI sufferers have some degree of hearing loss at much higher frequencies and hence cannot be considered a distinct group. If hearing loss alone were sufficient for abnormal slow wave activity, those with no TI and hearing loss would also exhibit abnormally enhanced slow wave activity. Based on the results of our masking study, it may be that slow-wave delta/theta activity is a neurological correlate of TI. In this respect, it is plausible that a reduction of this abnormal activity might signify a reduction in the level or perceived severity of TI and could potentially be used as a valuable indicator of the course of TI treatment.
